# Development of High-Internal-Phase Pickering Emulsions Stabilized by Soy Protein Isolate and Sodium Alginate as Innovative Fat Replacers for Emulsified Sausages

**DOI:** 10.3390/foods15081294

**Published:** 2026-04-09

**Authors:** Zhi Wang, Xuefei Wang, Xin Li, Chao Zhang, Fangda Sun, Qian Chen, Qian Liu, Baohua Kong, Haotian Liu

**Affiliations:** College of Food Science, Northeast Agricultural University, Harbin 150030, China; wangzhi9837@163.com (Z.W.); neauwangxuefei@163.com (X.W.); lixin200106062025@163.com (X.L.); zhangc323@hotmail.com (C.Z.); sunfangda@163.com (F.S.); chenqianego7@126.com (Q.C.); liuqian@neau.edu.cn (Q.L.)

**Keywords:** fat substitution, high-internal-phase Pickering emulsion, emulsified sausages, quality characteristic

## Abstract

In this study, vegetable oil-based high-internal-phase Pickering emulsions (HIPPEs) were formulated from soy protein isolate and sodium alginate, and the effects of different replacement ratios (20–100%) of pork back fat on the quality of emulsified sausages were investigated. With the increase in the fat replacement ratio, cooking loss, released fat, and lipid oxidation significantly decreased (*p* < 0.05). Similarly, as the replacement ratio rose, L*-values, pH and springiness increased, while a*-values, hardness, cohesiveness, and chewiness showed a significant decrease. The reformulated sausages exhibited superior slice compactness, a macroscopic trait corroborated by the dense network structure observed via microstructural analysis. Electronic nose and electronic tongue measurements indicated that the inclusion of HIPPEs modulated both the aroma profiles and taste attributes of the emulsified sausages. Moreover, although differences were observed in some sensory attributes and flavor characteristics, all formulations with HIPPEs remained within an acceptable sensory range.

## 1. Introduction

Emulsified meat products serve as a major source of essential nutrients and constitute a substantial portion of the modern human diet [1]. The production of these products generally requires the addition of 20–30% animal fat to achieve desirable functional and sensory properties [2]. However, animal fat contains high levels of saturated fatty acids. High dietary intake of saturated fatty acids is closely linked to an elevated risk of various chronic conditions, including obesity, atherosclerosis, and hypercholesterolemia, which are harmful to human health [3]. Indeed, the World Health Organization advocated as early as 2013 for the consumption of meat products with lower levels of animal fat or those enriched in polyunsaturated fatty acids. Heightened awareness of dietary health has driven a shift in consumer preferences toward meat products with more desirable fatty acid profiles. Nevertheless, simply lowering animal fat content often compromises the sensory quality and technological performance of meat products [4]. As a result, various technological strategies have been widely investigated in both the meat and food industries to reduce animal fat content, with the goal of developing healthier and more nutritious alternatives [5].

Recently, substantial research has focused on the use of protein- or polysaccharide-based ingredients as fat substitutes [6]. While these fat substitutes can mimic the appearance of animal fat, the absence of fat can adversely affect the flavor of the product, thereby reducing the satisfaction of consumers [7]. Researchers have explored strategies to replace animal fats with liquid vegetable oils, including emulsion gels, oleogels, microencapsulation, and lipid modification. Although these fat replacement strategies can be effective, they may also introduce specific quality challenges [8]. Nevertheless, certain substitutes have proven capable of lowering caloric density while maintaining the desirable texture and sensory properties that are often lost when animal fat is reduced [9].

High-internal-phase Pickering emulsions (HIPPEs) are highly concentrated emulsion systems stabilized by colloidal particles, typically characterized by an internal dispersed phase volume fraction exceeding 74%. Driven by the strong anchoring of these particles at the oil–water interface, HIPPEs form a rigid interfacial layer that serves as a robust barrier against droplet coalescence. This unique structure grants HIPPEs semi-solid, gel-like rheological characteristics, making them capable of enhancing flavor complexity and sensory depth in food matrices [10]. Structurally, animal adipose tissue is primarily composed of adipocytes, with cytoplasm containing fat droplets. A small amount of cytoplasm is squeezed to the cell periphery, resulting in flat, elliptical, or polygonal cell shapes that resemble the oil droplet distribution characteristic of HIPPEs [11]. Therefore, HIPPEs hold promise as fat substitutes in meat products.

Sausages constitute a distinctive and highly complex colloidal system in which fat serves not only as an energy source but also as a critical contributor to structural integrity, lubrication, juiciness, and overall sensory acceptance. However, much of the existing research on HIPPEs in comminuted meat products tends to focus on simpler models, such as patties, or fails to address the specific challenges posed by sausage processing, including intense mechanical shearing during chopping, thermal processing schedules, and long-term storage stability under refrigeration. To bridge this gap, researchers have begun evaluating the performance of HIPPEs in various food models with promising results. For instance, Zheng et al. [12] developed a HIPE by complexing whey protein isolate with octenyl succinic anhydride (OSA) starch and used it as a fat replacer in cookies, achieving sensory qualities comparable to those of margarine-based versions. Liu et al. [13] incorporated codfish protein- and casein-stabilized HIPEs as lard substitutes in fish cake, resulting in improved juiciness and tenderness while maintaining gel strength similar to the control. Badar et al. [14] demonstrated that replacing 50% of the fat with glycerol diester-based HIPPEs derived from flaxseed improved the nutritional profile of beef burgers while also enhancing sensory and textural attributes. Similarly, Wang et al. [15] applied a protein-stabilized HIPPEs from *Prinsepia utilis Royle* as an animal fat replacer in low-fat meatballs, which improved texture and exhibited superior water-holding capacity than formulations containing free fat or lard.

The selection of appropriate interfacial stabilizers critically influences the architectural integrity and rheological characteristics of HIPPEs. Our preliminary research proved that thermal treatment can transform soy protein isolate (SPI) and sodium alginate (SA) into effective Pickering particles, capable of stabilizing internal oil phases as high as 80% with exceptional viscosity and plasticity [16]. However, unlike coarsely minced patties, emulsified sausages undergo intense high shear chopping and rely on a heat-induced protein gel network. Therefore, this study explores the structure–function relationship of SPI-SA HIPPEs within this demanding environment. We specifically investigated whether the SPI-SA synergy could withstand severe mechanical processing and how these particles perform as structural fillers within a complex meat emulsion. By evaluating their microstructural and physical properties, this work aims to provide a better understanding of HIPPEs in complex meat systems. Hence, this work aims to engineer nutritionally enhanced emulsified sausages by substituting pork fat with these HIPPE systems. Specifically, a direct weight-based substitution of the fat ingredient was applied to investigate its practical impact on the physicochemical, structural, and sensory properties of the meat matrix. The cooking loss, released fat, proximate composition, and physicochemical and quality characteristics of emulsified sausages formulated with different fat replacement ratios were investigated. In addition, electronic nose, electronic tongue, and sensory analyses were conducted to comprehensively evaluate flavor and consumer acceptability.

## 2. Materials and Methods

### 2.1. Materials

Soy protein isolate (SPI, protein ≥ 90%) was sourced from Shandong Yuwang Ecological Food Co., Ltd. (Yucheng, China). Sodium alginate (SA, purity ≥ 98%, without added gelling salts) was supplied by Yuanye Biotechnology Co., Ltd. (Shanghai, China). Sunflower oil was purchased from Jiusan Food Co., Ltd. (Harbin, China). Post-rigor lean pork meat and pork backfat were purchased from Beidahuang Meat Corporation (Harbin, China), kept on ice during transportation to the laboratory and used on the same day. Collagen casing (18 mm in diameter) was purchased from Shenguan Collagen Group Co. Ltd. (Wuzhou, China). Sodium chloride was purchased from China Salt Industry Group Co., Ltd. (Shanghai, China). Sodium nitrite, composite phosphate and sodium erythorbate were purchased from Yiren Food Additive Company (Harbin, China). Spices were all purchased from Chunheyuan Food Co., Ltd. (Taizhou, China).

### 2.2. Preparation of HIPPEs

The HIPPEs used in this study were prepared according to our previously described method [16]. SPI (20 mg/mL) and SA (20 mg/mL) were each dispersed in distilled water and stored at 4 °C overnight to ensure complete hydration. Equal volumes of the SPI and SA solutions were then mixed and incubated in a water bath at 95 °C for 30 min. The resulting complex was freeze-dried to obtain SPI-SA colloidal particles. This freeze-drying step was essential to allow for precise control over the final particle concentration in the aqueous phase. These particles (20 mg/mL) were subsequently re-dispersed in distilled water, after which sunflower oil was added to achieve an oil-phase volume fraction 75%. The mixture was homogenized at 13,000 rpm for 3 min using a high-shear homogenizer (IKA T20 Basic, Staufen, Germany) to form the HIPPEs.

### 2.3. Fabrication of Emulsified Sausages

The basic formulations of emulsified sausages are presented in Table 1. Six different sausage formulations were designed: control (25% pork backfat), H-20 (20% pork backfat & 5% HIPPEs), H-40 (15% pork backfat & 10% HIPPEs), H-60 (10% pork backfat & 15% HIPPEs), H-80 (5% pork backfat & 20% HIPPEs), and H-100 (25% HIPPEs). It is important to note that this formulation design was based on a direct one-to-one weight replacement of the pork backfat with HIPPEs.

The emulsified sausages used in this study were prepared following the method outlined by Zhao et al. [17] with some modifications. Visible connective tissues were removed from the fresh raw meat, after which the lean pork and backfat were cut into pieces and grated. Lean pork, salt, nitrite, composite phosphates, and half of the ice were chopped at high speed for 3 min. Spices and other auxiliary ingredients were then added and chopped for an additional 3 min. Subsequently, pork backfat, HIPPEs, and the remaining ice were added and chopped for another 3 min. Sodium erythorbate was incorporated just before completing the chopping process. Collagen casings with an 18 mm diameter were filled with the prepared meat batter using a stuffer and coiled on racks for drying. The emulsified sausages were cooked for 30 min at approximately 75 °C to ensure the internal core temperature reached at least 72 °C, complying with standard food safety protocols for pasteurization. Then, the emulsified sausages were rapidly cooled in ice water and stored in a refrigerator at 4 °C. Moreover, the entire production process was performed in triplicate, with three independent batches of sausages prepared to ensure reproducibility.

### 2.4. Cooking Loss and Released Fat

The cooking loss (%) and released fat were determined in triplicate for each batch, following the method reported by Lu et al. [18].

### 2.5. Proximate Composition

Moisture, protein, and ash contents were analyzed in triplicate for each batch, following the AOAC [19] official methods.

### 2.6. Instrumental Color, pH, and Textural Profile Analysis

Color parameters were obtained using a ZE-6000 colorimeter (Nippon Denshoku, Kogyo Co., Ltd., Tokyo, Japan), based on the procedure outlined by Nacak et al. [20].

For pH analysis, 2 g of the sausage sample was homogenized with 18 mL of distilled water. The mixture was left to stand for 30 min and filtered prior to measurement.

Texture analysis of raw meat batters was conducted using a TA-XT plusC texture analyzer (Stable Micro Systems Co., Ltd., Godalming, UK) following the procedure outlined by Hovjecki et al. [21]. A back-extrusion device (A/BE) probe was employed to record parameters including liquidity, uniformity, consistency, and viscosity.

Texture profile analysis (TPA) of the sausages was performed based on the method described by Nacak et al. [20]. Briefly, the cooked sausages were cut into cylinders with a height of 20 mm. TPA was conducted using a TA-XT plus texture analyzer equipped with a P/50 probe. The operating parameters were set as follows: a test speed and post-test speed of 1.0 mm/s, a compression ratio of 50%, a trigger force of 5 g, and a 5.0 s interval between the two compressions.

### 2.7. TBARS of Emulsified Sausages

Lipid oxidation was assessed by quantifying 2-thiobarbituric acid reactive substances (TBARS), following the protocol of Jin, Choi, & Kim [22] with minor modifications. Briefly, 2 g of the sausage sample was combined with 3 mL of thiobarbituric acid (TBA) and 17 mL of trichloroacetic acid (TCA)-HCl solution. The mixture was vortexed, heated in a boiling water bath for 30 min, and subsequently cooled. A 4 mL aliquot of the reaction mixture was combined with 4 mL chloroform and centrifuged at 1000× *g* for 10 min. The absorbance of the upper layer was recorded at 532 nm. TBARS values were calculated and expressed as mg malondialdehyde (MDA)/kg of sausage. The TBARS was performed with six replicates per treatment for each batch.

### 2.8. Scanning Electron Microscopy

The microstructure of emulsified sausages was measured with an S-3400N scanning electron microscope equipped (Hitachi, Japan) following the method of Nan et al. [23].

### 2.9. Electronic Nose and Electronic Tongue Analysis

Electronic nose measurements were performed according to the method described by Zhao et al. [17]. Individual sensors responded differently to various classes of volatile compounds, allowing overall aroma profiles to be distinguished from the combined sensor data. Taste profiles of the emulsified sausages were evaluated using a TS-5000Z electronic tongue (Insent Inc., Atsugi-shi, Japan) equipped with artificial lipid membrane sensors. Briefly, 30 g of minced sausage was blended with 150 mL of distilled water to extract taste-active compounds. The mixture was incubated at 40 °C for 30 min and subsequently centrifuged at 5000× *g* for 10 min. The supernatant was filtered, and the resulting filtrate was subjected to analysis.

### 2.10. Sensory Evaluation

Sensory evaluation was performed by previously reported methods [23]. Sensory evaluation was performed by a trained panel of 16 judges (8 males and 8 females), comprising faculty members and graduate students with prior experience in meat product assessment. Three training sessions were held over a two-week period. The initial two sessions focused on generating descriptors for interior color, uniformity, juiciness, and flavor. In the final session, reference samples were used to calibrate panelists and establish consensus on descriptor definitions. Because the modified sausages exhibited slight visual differences due to fat replacement, panelists were explicitly trained to evaluate each attribute independently to prevent any visual bias or halo effect. Attributes were evaluated on a 7-point intensity scale, ranging from 1 (not perceived) to 7 (extremely intense). For evaluation, samples were cut into 2.0 cm cubes, presented on white ceramic plates, and labeled with random 3-digit codes to ensure blinding. The samples were presented to the panelists in a randomized, balanced order to minimize carry-over effects. All evaluations were conducted in individual sensory booths under standard white lighting. Informed consent for participation was obtained from all subjects involved in the study.

### 2.11. Statistical Analysis

Data were analyzed using Statistix 8.1 (Analytical Software, St. Paul, MN, USA) and are presented as means ± standard error (SE). Data were subjected to a one-way analysis of variance (ANOVA), and subsequently, differences between means were evaluated using Tukey’s multiple comparison test, with significance defined at *p* < 0.05.

## 3. Results and Discussion

### 3.1. Proximate Composition

Substituting pork backfat with HIPPEs led to significant differences in the moisture and fat contents of emulsified sausages (Table 2). As HIPPE levels increased, fat content decreased while moisture content rose (*p* < 0.05), largely due to the higher water and lower fat contents of HIPPEs compared to pork backfat. The gel-like network of HIPPEs also enhanced water-binding capacity. The trend reflects a successful reduction in caloric density, as the high-fat animal tissue was replaced by a structured emulsion containing an aqueous phase. Furthermore, the solid-like rheological properties of HIPPEs ensured that the additional moisture remained trapped within the emulsion droplets during the initial mixing phase, preventing phase separation before thermal gelation. This observation aligns with the findings of Zhao et al. [24], who reported that quinoa protein-stabilized emulsions used as fat substitutes significantly increased the moisture content of frankfurters. Protein and ash contents did not differ significantly across all formulations (*p* > 0.05). This indicates that the SPI-SA composite particles contributed a negligible protein fraction, insufficient to alter the protein and mineral profiles dominated by the meat matrix. Similarly, Li et al. [25] found that emulsions stabilized by zein and carboxymethyl dextrin did not significantly alter these components in sausages.

### 3.2. Cooking Loss and Released Fat of Emulsified Sausages

**Figure 1:**
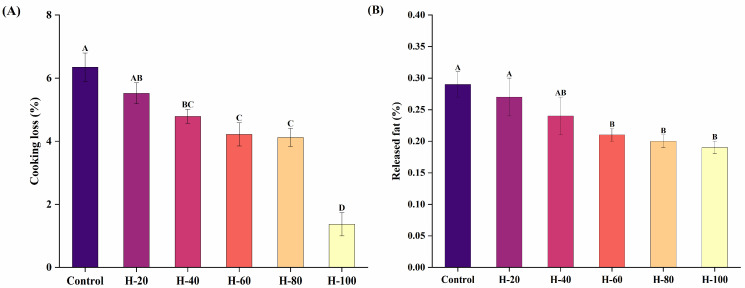
Changes in cooking loss (**A**) and released fat (**B**) of emulsified sausages using HIPPEs as fat replacers. Data are expressed as mean ± standard error. Different letters (A–D) indicate significant differences (*p* < 0.05).

Cooking loss is a key indicator for evaluating reformulated sausage products, as it reflects their ability to retain water and lipids during thermal processing [26]. As shown in Figure 1A, cooking loss of emulsified sausages decreased significantly with increasing levels of fat substitution (*p* < 0.05). The lowest cooking loss was observed in the H-100 treatment groups compared with all other formulations (*p* < 0.05). Similarly, Chen, Wang, Fei, Tan, & Cheng [27] found that the cooking loss of sausages decreased by 67% when 100% of animal fat was replaced with HIPPEs. This decrease in cooking loss is linked to enhanced water and lipid retention, which in turn boosts final product yield and sensory quality. The incorporation of HIPPEs leads to the formation of a stable meat system, likely due to their excellent thermal stability. Furthermore, this improved water-binding capacity can be explained by protein–lipid interactions. During chopping and heating, myofibrillar proteins unfold and expose their hydrophobic groups. These unfolded meat proteins can then interact with the SPI-SA particles at the HIPPEs interface through hydrophobic forces and physical entanglement. Such interfacial cross-linking helps build a denser three-dimensional gel network. As a result, the stronger capillary forces better retain moisture and lipids, directly reducing cooking loss. When HIPPEs replace animal fat in emulsified sausages, the emulsion undergoes a double emulsification process, promoting a uniform incorporation throughout the meat protein matrix and further minimizing cooking losses [18].

As shown in Figure 1B, the amount of released fat in the emulsified sausages decreased significantly (*p* < 0.05) with higher HIPPE substitution levels, mirroring the trend observed in cooking loss. At H-100, released fat reached its lowest level relative to the control group (*p* < 0.05), suggesting that the fat was effectively encapsulated by SPI-SA particles. These findings demonstrate that HIPPEs are efficiently emulsified and evenly dispersed throughout the sausage matrix, with SPI-SA particles binding tightly to oil droplet surfaces and interacting with meat proteins. This enhanced stability stems from the irreversible adsorption of SPI-SA particles at the oil–water interface, which forms a rigid mechanical barrier that prevents droplet coalescence and subsequent oil leakage under thermal stress. Moreover, the superior thermal stability of HIPPEs enhances the structural integrity of the meat matrix during thermal processing. Consistent with these findings, Kim, Yong, Jung, Kim, & Choi [28] demonstrated that substituting a portion of pork backfat with emulsified grape seed oil enhanced the emulsion stability of meat batters. In general, HIPPE supplementation markedly lowered the fat release of emulsified sausages, primarily due to their high thermal stability.

### 3.3. Flow Characteristic of Raw Meat Batter

The A/BE probe is well suited for characterizing the mechanical behavior of semi-fluid or soft materials such as raw meat batters. Each sample was loaded into a cylindrical container and subjected to compression with a circular disc probe. During compression, the plunger descends into the sample, forcing it to flow upward and radially around the plunger edge. This process generates a force–time curve that reflects the deformation resistance and flow characteristics of the material. By analyzing parameters such as deformation behavior, resistance force, and pressure variation, the back-extrusion test provides valuable insights into the mechanical and flow-related properties of the sample [29]. The liquidity of the raw meat batter was obtained using this back-extrusion test. The initial slope of the force–time curve, representing the sample’s initial resistance to deformation, was used to quantify liquidity. A lower slope value indicated higher liquidity, whereas a higher slope reflected lower liquidity. Similarly, smaller uniformity values corresponded to better internal homogeneity of the sample.

The liquidity, uniformity, consistency, and viscosity of raw meat batters prepared with HIPPEs as fat replacers are presented in Table 3. Relative to the control group, incorporating HIPPEs significantly improved both the fluidity and internal uniformity of the raw meat batter (*p* < 0.05). This improvement stems from the replacement of solid, irregular fat particles with soft, deformable lipid droplets from HIPPEs, which facilitated easier flow and created a more homogeneous structure under compression [30]. Meanwhile, substitution with HIPPEs notably reduced the consistency and viscosity (*p* < 0.05). Similarly, Liu et al. [31] indicated that replacing animal fat with vegetable-based systems leads to lower viscosity and a weakened structure of meat paste. This decline is primarily attributed to the soft matter characteristics of HIPPEs, which lack the structural rigidity of solid pork fat. Their inclusion as a soft filler interferes with the protein matrix to produce a more compliant and less viscous batter. Moreover, samples containing HIPPEs showed significantly reduced cohesiveness compared with the control (*p* < 0.05). This reduction suggests a weakened internal binding within the meat batter, likely stemming from a partial disruption of the continuous protein matrix by the incorporated lipid phase. In summary, replacing pork backfat with HIPPEs effectively modified the flow-related texture characteristics of raw meat batters, resulting in softer, less viscous, and more processable formulations.

### 3.4. Instrumental Color, pH, and Textural Profile Analysis

Color constitutes a critical quality attribute in meat products. The instrumental color values of the emulsified sausages using HIPPEs as fat replacers are shown in Table 4. Increasing HIPPE substitution levels led to a significant increase in L*-values (lightness) and a concurrent decrease in a*-values (redness) compared to the control (*p* < 0.05). This trend aligns with the findings of Galvão, Costa, Santos, Pollonio, & Hubinger [32]. The reduction in a*-values is due to the dilution effect of the milky-white HIPPEs replacing the pigmented pork backfat. Conversely, the rise in L*-values is likely attributed to the enhanced light-scattering properties of the fine oil droplets distributed within the HIPPE matrix, mirroring the findings of Lu et al. [18] in pre-emulsified oil-based sausages. However, our results contrast with those of Wang et al. [33], who reported a significant decrease in L*-values and an increase in a*-values upon fat substitution. Their research indicated that reducing the fat content limits the density of surface oil droplets, thereby diminishing light reflection and decreasing L*-values. Furthermore, they attributed the rise in a*-values to the reduced abundance of white solid fat particles, which originally masked the redness of the meat matrix. Such discrepancies are likely due to variations in oil composition, emulsion droplet size, and formulation ingredients across studies. Overall, HIPPE incorporation significantly enhanced the lightness of the sausages, altering the visual profile and potentially influencing consumer acceptance.

The pH value constitutes a critical quality parameter in emulsified meat products, as it directly dictates the solubilization of myofibrillar proteins and the stability of the batter. The functional properties of emulsified sausages are generally optimized when the pH is between 6.5 and 7.6. As shown in Table 4, the pH values ranged from 6.60 to 6.73, remaining within the optimal range. Notably, the incorporation of HIPPEs induced a slight but statistically significant reduction in pH compared to the control (*p* < 0.05). This observation aligns with Badar et al. [14], who reported a similar pH decline in beef burgers with increasing fat replacement levels.

Texture is a critical determinant of consumer acceptance, encompassing the perception of bite, chewiness, and mouthfeel. As presented in Table 4, fat substitution resulted in a significant reduction in hardness, cohesiveness, and chewiness compared with the control (*p* < 0.05). Specifically, the control sample exhibited the highest hardness, whereas the 100% replacement group demonstrated the lowest, consistent with previous reports that fat substitution by emulsions reduces sausage hardness [34]. Notably, a significant decline in cohesiveness was observed only when the substitution ratio reached 100% (*p* < 0.05). The observed reduction in chewiness implies that the fat-substituted sausages required less mechanical effort for mastication. The decline in textural parameters is primarily driven by two factors. First, despite their high viscoelasticity, the HIPPEs possess lower intrinsic hardness than crystalline pork backfat. Consequently, they act as soft fillers that reduce the mechanical resistance of the gel network. Second, the strong water-binding capacity of HIPPEs may alter water distribution within the sausage matrix, which can interfere with protein–protein interactions and disrupt the continuity of the gel network. Collectively, these effects contribute to a softer texture and reduced structural integrity, particularly at high substitution levels. Contrarily, Gao et al. [35] reported that a high level of HIPPE substitution significantly enhanced the texture of meat patties, attributing the increased hardness to reduced oil and water loss that reinforced the coarse matrix. The discrepancy likely stems from the fundamental structural differences between meat patties and emulsified sausages.

### 3.5. TBARS of Sausages

**Figure 2:**
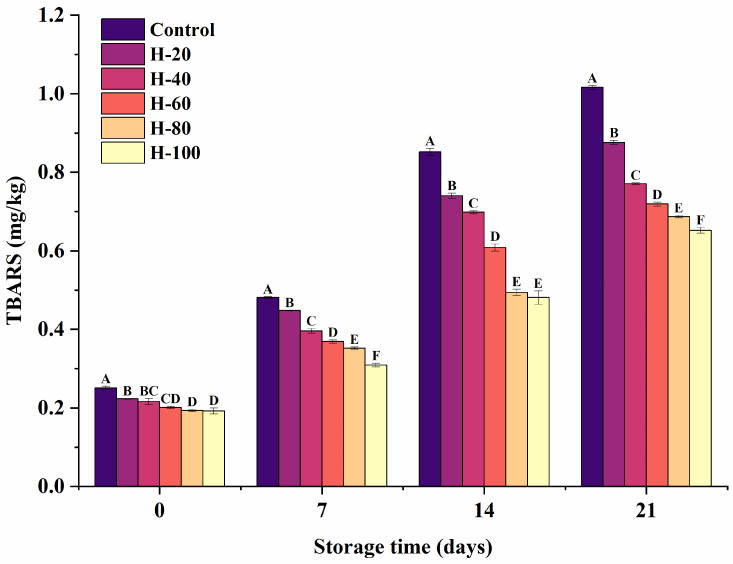
Changes in TBARS of emulsified sausages using HIPPEs as fat replacers stored at 4 °C for various periods. Data are expressed as mean ± standard error. Different letters (A–F) indicate significant differences (*p* < 0.05).

While moderate lipid oxidation plays a role in enhancing flavor development in meat products, excessive oxidation generates off-odors that detrimentally affect consumer acceptance. Figure 2 illustrates the temporal changes in TBARS values for emulsified sausages with varying HIPPE substitution levels during 21 days of refrigerated storage at 4 °C. As expected, TBARS values rose in all samples during storage, indicating the progressive accumulation of secondary lipid oxidation products. This trend aligns with earlier observations reported in frankfurters [36], reaffirming that lipid oxidation is an inevitable consequence of prolonged meat storage. Sausages formulated with HIPPEs exhibited significantly reduced TBARS values compared to the control (*p* < 0.05), with the inhibitory effect becoming more pronounced at higher substitution levels. This enhanced oxidative stability is likely attributed to the dense interfacial layer formed by SPI-SA composite particles, which effectively encapsulates the oil droplets. Furthermore, the interaction between these particles and the protein matrix generates a compact physical barrier, limiting oxygen diffusion and reducing lipid accessibility to oxidative agents. Moreover, the emulsified lipids within the Pickering structure are shielded from pro-oxidants in the meat matrix, unlike the bulk pork fat in the control, which is more exposed. These findings align with the observations of Zhang et al. [37] in fat-reduced pork batter. Beyond the interfacial shielding effect, they proposed that the increased viscosity of the Pickering emulsion matrix further suppresses oxidation by restricting the mobility of free radicals and metal ions. The generally accepted sensory threshold for TBARS in meat products is approximately 1.0 mg MDA/kg, beyond which rancid flavors become noticeable. Notably, TBARS values in all HIPPE-treated groups remained below this threshold throughout storage, whereas the control group exceeded the limit after 21 days. These results highlight the superior oxidative stability conferred by the HIPPE system, further supporting its potential as a functional fat replacer in meat products.

### 3.6. Scanning Electron Microscopy (SEM)

**Figure 3:**
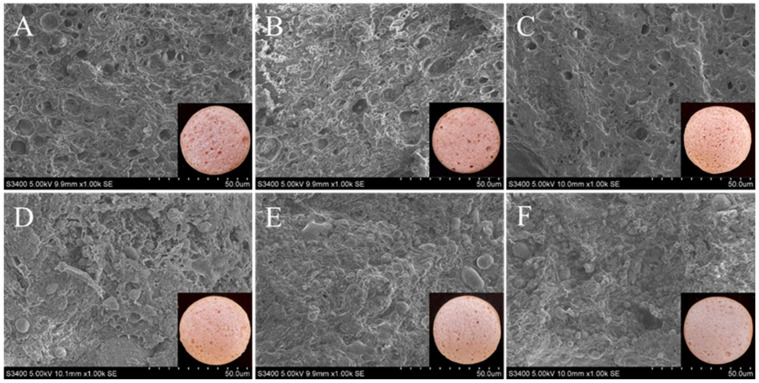
SEM and apparent images of emulsified sausages using HIPPEs as fat replacers. (**A**): emulsified sausages prepared with100% pork backfat; (**B**–**F**): emulsified sausages prepared by substituting 20%, 40%, 60%, 80% and 100% pork backfat with HIPPEs.

Microstructural analysis via SEM is pivotal for elucidating the relationship between formulation, processing, and quality in emulsified sausages. Specifically, this technique reveals how fat substitutes interact with muscle proteins to modulate texture. As shown in Figure 3, the control samples displayed the typical heat-induced emulsion gel morphology characterized by a heterogeneous, honeycomb-like network formed through protein matrix aggregation. The voids within this network correspond to areas originally occupied by water and fat globules that were removed during dehydration [38]. In the native state, salt-soluble proteins stabilize fat globules through amphiphilic interactions, forming an oil-in-water emulsion embedded within a robust three-dimensional protein network. With increasing HIPPE content, however, the network became progressively more homogeneous, exhibiting greater compactness and smaller cavity size. This structural refinement can be attributed to two mechanisms. First, the smaller droplet size of HIPPEs facilitates more uniform dispersion and integration within the meat batter compared with pork backfat [33]. On the other hand, SPI-SA composite particles may facilitate myosin anchoring at the oil–water interface, thereby preserving salt-soluble proteins for gel network reinforcement. Similarly, Qi et al. [30] found that HIPPEs could be uniformly distributed throughout the sausage interior as fine droplets, resulting in the formation of a more compact protein gel network with reduced pore size. Such microstructural observations corresponded well with the enhanced emulsion stability and reduced cooking loss. This structural modification toward a denser network was confirmed by macroscopic analysis, where the H-100 treatment exhibited the greatest compactness.

### 3.7. Electronic Nose Analysis

**Figure 4:**
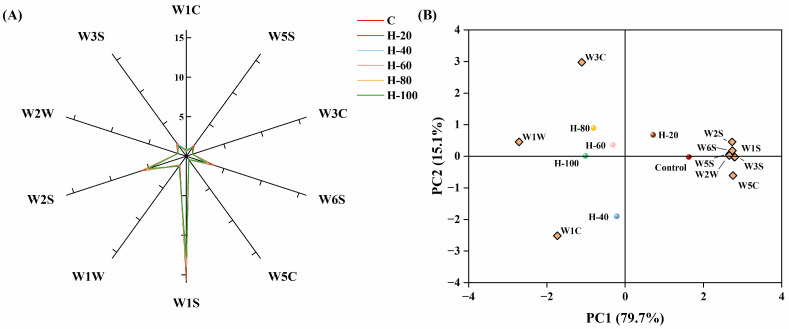
Response value (**A**) and principal component analysis (**B**) of the electronic nose data for emulsified sausages using HIPPEs as fat replacers.

The electronic nose is extremely responsive to aroma characteristics, and even minor variations in volatile compounds can lead to noticeable differences in sensor responses. As illustrated in Figure 4A, the W1S, W2S, and W6S sensors exhibited the strongest signals among all channels, indicating comparatively higher levels of alcohols, sulfur-related volatiles, and other aromatic compounds in the sausage samples. In particular, the response intensities of W1S, W2S, W3S, W5S, and W6S were consistently higher in the control group than in the HIPPE-substituted samples, suggesting that pork backfat contributed to a greater abundance of methyl, alcohols, aldehydes, ketones, long-chain alkanes, hydrides, and sulfides [17]. Such disparity likely stems from the characteristic lipid oxidation and thermal degradation products of animal fats, which are fundamental to the traditional “meaty” aroma of emulsified sausages. Moreover, increasing the substitution ratios led to elevated responses in the W1C and W1W sensors, implying a higher presence of aromatics and sulfur-organic volatiles in sausages where pork backfat was progressively replaced. The upward trend suggests that while the animal-derived volatiles decreased, the incorporation of plant-based HIPPEs introduced a new set of volatile precursors or facilitated the release of specific aromatic compounds from the vegetable oil phase.

According to the principal component analysis (PCA) distribution (Figure 4B), the control and H-20 groups were located along the positive half-axis of PC2, correlating with sensors W1S, W2S, W3S, W5S, W6S, W1C, W5C and W2W. In contrast, the H-40, H-60, H-80, and H-100 treatments were positioned on the negative half-axis of PC2, correlated with W1C, W3C, and W1W. PC1 and PC2 together accounted for 94.8% of the total variance, indicating that the PCA model effectively distinguished odor profiles among treatments. The separation pattern indicated that higher HIPPE substitution levels reduced aldehydes and sulfide volatiles while enhancing responses associated with aromatics, alcohols, and nitrogen-containing compounds. These changes were primarily attributed to the reduction in pork backfat, which limits the lipid oxidation products. Additionally, the incorporation of HIPPEs modulates protein–lipid interactions and influences the release of Maillard-derived volatiles. Overall, partial substitution (H-20 and H-40) exerted only minor effects on odor characteristics, whereas higher substitution levels produced more distinct shifts in the volatile profile of emulsified sausages.

### 3.8. Electronic Tongue Analysis

**Figure 5:**
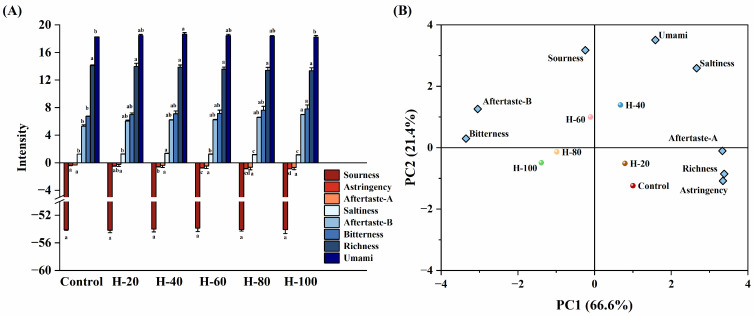
Response value (**A**) and principal component analysis (**B**) of the electronic tongue data for emulsified sausages using HIPPEs as fat replacers. Different letters (a–d) indicate significant differences (*p* < 0.05).

The electronic tongue serves as a biomimetic instrument capable of objectively quantifying variations in the taste profile of food matrices [39]. As shown in Figure 5A, HIPPE substitution influenced several taste attributes of the emulsified sausages. The complete replacement group (H-100) exhibited the highest intensities of bitterness and aftertaste-B, significantly exceeding those of the control (*p* < 0.05). Conversely, astringency intensity decreased significantly with increasing HIPPE substitution levels, with the control group exhibiting the highest astringency. Umami and Saltiness intensities peaked significantly in the 40% substitution level compared with the control (*p* < 0.05). Most importantly, no significant differences were observed among treatments for richness, sourness, and aftertaste-A (*p* > 0.05). The absence of variation in richness suggests that HIPPEs successfully reproduced the sensory contribution of pork backfat, thereby maintaining the perceived richness of the sausages even at 100% substitution.

PCA results are presented in Figure 5B. PC1 and PC2 accounted for 66.6% and 21.4% of the variance, respectively, yielding a cumulative contribution of 88%. The control, H-20, and H-40 treatment groups were distributed on the positive half-axis of PC1 and were associated with richness, saltiness, astringency, aftertaste A, and umami. The H-60, H-80, and H-100 treatment groups were positioned on the negative half-axis of PC1, correlating with bitterness, aftertaste B, and sourness. This indicates that the overall taste profiles of the control, H-20, and H-40 groups were similar, whereas higher substitution levels progressively shifted the sensory profile toward increased bitterness and sourness, accompanied by reduced astringency.

### 3.9. Sensory Evaluation

Since consumer perception ultimately determines the market success of reformulated meat products, sensory evaluation serves as a crucial method for assessing the acceptability of emulsified sausages [40]. In this work, overall product quality was assessed through sensory evaluation of interior color, uniformity, flavor, and juiciness (Table 5). Flavor scores were comparable among the control, H-20, and H-40 groups, indicating that partial substitution of fat with HIPPEs does not negatively influence product flavor. This is a favorable finding, as flavor strongly influences consumer purchasing decisions. Color is a paramount sensory descriptor for meat products, profoundly influencing consumer perception and purchasing decisions. Increasing HIPPE substitution levels significantly reduced the internal color score, reaching the lowest value at the H-100 group (*p* < 0.05). This visual phenomenon likely stems from the inherently pale color of HIPPEs, which aligns perfectly with the instrumental color analysis demonstrating increased L* and decreased a*. Additionally, the uniformity and juiciness score initially increased with higher HIPPE substitution levels. This sensory perception is highly consistent with the reduced cooking loss and the enhanced instrumental texture parameters. These improvements may be associated with the more compact gel network structure formed through interactions between myofibrillar proteins and HIPPEs. Overall, all sensory attribute scores surpassed 5 points, indicating that substituting pork backfat with HIPPEs preserved sausage quality at levels consumers would find acceptable. These findings further confirm that HIPPEs function effectively as fat substitutes without compromising consumer satisfaction. Given their ability to preserve flavor and improve texture, HIPPEs show great promise for developing healthier, reduced-fat meat products that meet consumer demands for both improved nutritional quality and enhanced sensory characteristics.

## 4. Conclusions

This study demonstrated that HIPPEs stabilized by SPI-SA particles are effective fat substitutes in emulsified sausages. Substituting pork backfat with HIPPEs significantly reduced cooking loss, fat exudation, and lipid oxidation, thereby improving processing stability. Microstructural analysis confirmed that HIPPE incorporation facilitated the development of a denser, more continuous protein network with fewer voids, contributing to enhanced structural integrity. Although excessive substitution altered texture and aroma profiles, sensory evaluation confirmed that all formulations remained consumer-acceptable. Collectively, these findings suggest that HIPPEs stabilized by SPI-SA particles can serve as a promising technological strategy for replacing animal fat in meat products without compromising overall physical and sensory quality. This approach provides the meat industry with a practical method to develop reduced-animal-fat formulations. However, to fully separate the structural benefits of the HIPPE matrix from the simple effects of replacing animal fat with vegetable oil, future studies should include unstructured oil and conventional emulsion controls. Additionally, evaluating long-term shelf-life stability and detailed descriptive sensory profiles is necessary to further optimize these formulations for commercial application.

## Figures and Tables

**Table 1:** Formulations of emulsified sausages with HIPPEs as a fat substitute at different ratios.

	Control	H-20	H-40	H-60	H-80	H-100
Lean meat (g)	1000	1000	1000	1000	1000	1000
Pork backfat (g)	500	400	300	200	100	0
HIPPEs (g)	0	100	200	300	400	500
Ice-water mixture (g)	500	500	500	500	500	500
Salt (g)	30	30	30	30	30	30
Compound phosphate (g)	8	8	8	8	8	8
White pepper powder (g)	6	6	6	6	6	6
Red bell pepper powder (g)	5	5	5	5	5	5
Ginger powder (g)	6	6	6	6	6	6
Nutmeg powder (g)	5	5	5	5	5	5
Monosodium glutamate (g)	1	1	1	1	1	1
Sodium nitrite (g)	0.15	0.15	0.15	0.15	0.15	0.15

Control: emulsified sausages prepared with 100% pork backfat; H-20, H-40, H-60, H-80, and H-100: emulsified sausages prepared by substituting 20%, 40%, 60%, 80% and 100% pork backfat with HIPPEs.

**Table 2:** Changes in proximate analysis of emulsified sausages using HIPPEs as fat replacers.

	Moisture (%)	Fat (%)	Protein (%)	Ash (%)
Control	58.15 ± 0.24 ^c^	20.18 ± 0.19 ^a^	15.95 ± 0.12 ^a^	1.81 ± 0.04 ^a^
H-20	58.62 ± 0.31 ^c^	19.91 ± 0.23 ^a^	15.89 ± 0.14 ^a^	1.79 ± 0.03 ^a^
H-40	59.33 ± 0.22 ^bc^	19.62 ± 0.16 ^ab^	15.87 ± 0.11 ^a^	1.82 ± 0.05 ^a^
H-60	59.87 ± 0.18 ^ab^	18.95 ± 0.15 ^bc^	15.76 ± 0.13 ^a^	1.80 ± 0.04 ^a^
H-80	60.56 ± 0.26 ^a^	18.59 ± 0.20 ^c^	15.69 ± 0.15 ^a^	1.78 ± 0.03 ^a^
H-100	61.04 ± 0.29 ^a^	18.36 ± 0.24 ^c^	15.70 ± 0.10 ^a^	1.81 ± 0.05 ^a^

Data are expressed as mean ± standard error. Different letters (a–c) indicate significant differences in the same column (*p* < 0.05).

**Table 3:** Changes in mechanical properties of uncooked meat batters using HIPPEs as fat replacers.

	Liquidity	Uniformity	Consistency	Viscosity
Control	61.95 ± 4.13 ^a^	527.20 ± 9.54 ^a^	5409.19 ± 271.86 ^a^	1072.16 ± 22.93 ^a^
H-20	59.31 ± 3.30 ^a^	497.16 ± 5.65 ^b^	5089.97 ± 112.75 ^b^	1028.86 ± 10.04 ^b^
H-40	54.24 ± 2.79 ^b^	472.54 ± 7.19 ^c^	4782.74 ± 109.89 ^c^	941.81 ± 34.12 ^c^
H-60	44.40 ± 2.03 ^c^	450.31 ± 2.90 ^d^	4520.56 ± 117.72 ^d^	882.23 ± 14.05 ^d^
H-80	40.51 ± 1.24 ^d^	435.78 ±5.37 ^e^	4231.10 ± 150.19 ^e^	810.50 ± 50.93 ^e^
H-100	36.88 ± 1.69 ^e^	406.79 ±6.17 ^f^	4185.16 ± 167.72 ^e^	798.17 ± 61.37 ^e^

Data are expressed as mean ± standard error. Different letters (a–f) indicate significant differences in the same column (*p* < 0.05).

**Table 4:** Changes in instrumental color, pH, and texture profile analysis of emulsified sausages using HIPPEs as fat replacers.

	Control	H-20	H-40	H-60	H-80	H-100
L*	62.29 ± 0.13 ^e^	63.15 ± 0.46 ^d^	64.08 ± 0.24 ^c^	66.46 ± 0.35 ^b^	67.49 ± 0.18 ^a^	67.63 ± 0.23 ^a^
a*	11.45 ± 0.30 ^a^	11.23 ± 0.29 ^a^	10.53 ± 0.60 ^b^	9.89 ± 0.37 ^c^	9.26 ± 0.43 ^d^	9.14 ± 0.16 ^d^
b*	18.01 ± 0.06 ^a^	18.04 ± 0.58 ^a^	18.10 ± 0.17 ^a^	18.16 ± 0.22 ^a^	18.22 ± 0.40 ^a^	18.35 ± 0.45 ^a^
pH	6.76 ± 0.03 ^a^	6.73 ± 0.01 ^ab^	6.65 ± 0.01 ^bc^	6.64 ± 0.02 ^c^	6.60 ± 0.02 ^c^	6.61 ± 0.01 ^c^
Hardness (N)	23.16 ± 0.11 ^a^	22.74 ± 0.08 ^ab^	22.45 ± 0.09 ^b^	21.26 ± 0.10 ^c^	21.11 ± 0.10 ^c^	20.96 ± 0.14 ^c^
Springiness	0.80 ± 0.01 ^c^	0.82 ± 0.01 ^bc^	0.85 ± 0.01 ^b^	0.90 ± 0.01 ^a^	0.90 ± 0.02 ^a^	0.92 ± 0.01 ^a^
Cohesiveness	0.71 ± 0.02 ^a^	0.69 ± 0.02 ^a^	0.64 ± 0.02 ^ab^	0.65 ± 0.02 ^ab^	0.64 ±0.01 ^ab^	0.59 ± 0.02 ^b^
Chewiness (N)	19.62 ± 0.52 ^a^	16.25 ± 0.89 ^b^	13.98 ± 1.01 ^bc^	12.51 ± 0.76 ^c^	11.49 ± 0.78 ^c^	12.80 ± 0.67 ^c^

Data are expressed as mean ± standard error. Different letters (a–e) indicate significant differences in the same row (*p* < 0.05).

**Table 5:** Changes in the sensory analysis of emulsified sausages using HIPPEs as fat replacers.

	Control	H-20	H-40	H-60	H-80	H-100
Interior color	6.78 ± 0.08 ^a^	6.75 ± 0.09 ^a^	6.61 ± 0.05 ^b^	6.45 ± 0.06 ^c^	6.04 ± 0.07 ^d^	5.34 ± 0.09 ^e^
Uniformity	4.83 ± 0.18 ^b^	4.96 ± 0.16 ^ab^	5.20 ± 0.11 ^ab^	5.29 ± 0.19 ^ab^	5.46 ± 0.07 ^ab^	5.50 ± 0.11 ^a^
Flavor	6.65 ± 0.15 ^a^	6.45 ± 0.22 ^a^	6.10 ± 0.23 ^a^	5.75 ± 0.24 ^b^	5.50 ± 0.25 ^b^	5.10 ± 0.20 ^c^
Juiciness	5.04 ± 0.14 ^e^	5.26 ± 0.15 ^e^	5.36 ± 0.16 ^d^	5.72 ± 0.17 ^c^	5.88 ± 0.18 ^b^	5.92 ± 0.18 ^a^

Data are expressed as mean ± standard error. Different letters (a–e) indicate significant differences in the same row (*p* < 0.05).

## Data Availability

The original contributions presented in this study are included in the article. Further inquiries can be directed to the corresponding authors.
